# Editorial: Addressing the effects of COVID-19 on rural areas in low and middle income countries

**DOI:** 10.3389/fpubh.2022.976978

**Published:** 2022-11-01

**Authors:** Masoud Yazdanpanah, Nadejda Komendantova, Love Ekenberg, Ahmed Al-Salaymeh

**Affiliations:** ^1^Department of Agricultural Extension and Education, Agriculture Sciences and Natural Resources University of Khuzestan, Mollasani, Iran; ^2^Department of Family, Youth, and Community Sciences, University of Florida, Gainesville, FL, United States; ^3^Advancing Systems Analysis Program, Cooperation and Transformative Governance Group, International Institute for Applied Systems Analysis, Laxenburg, Austria; ^4^Department of Computer and Systems Sciences, Stockholm University, Kista, Sweden; ^5^Department of Mechanical Engineering, School of Engineering, The University of Jordan, Amman, Jordan

**Keywords:** editorial, COVID-19, rural area, low and middle income, articles

Rural households are facing severe challenges while adapting to existing and emerging risks of various kinds, including the effects of unprecedented shocks that affect individuals and families in terms of lack of income, reduced consumption and the sale of assets. Health shocks, i.e., unpredictable diseases that undermine people's health status, are the most common unprecedented shocks and the most pressing causes of families falling into poverty. When an illness or injury impairs the health of a family member or causes the income of the family to be reduced or even lost, the family is faces high levels of vulnerability due to the costs of medical treatment as well as the lack of income caused by incapacity for work. Health shocks, together with economic ditto, thus place a heavy financial burden on families and are, de facto, one of the most important factors related to poverty in these regions.

Risk adaptation and coping has become necessary activities for families living in rural areas of low and middle income countries, but this is also taking a significant share of their income. Consequently, understanding these risks and related coping strategies is crucial for policymakers. This is also reflected in the Global Development Report entitled Risks and Opportunities, which examines how families can cope with the wide range of risks they face.

The focus of this special issue addresses the effects of COVID-19 on rural areas in the Low- and Middle - Income Countries. Several researchers submitted a significant number of high-quality papers for consideration in this special issue, which went through a rigorous peer-review process, with an acceptance rate of 50%. Finally, 16 quality contributions were published, and among them were the following papers:

- *COVID-19's Impact on China's Strategic Emerging Industries: An Observation of Policy Difficulties* studies the influence of the COVID-19 on R&D investment and foreign exchange development of China's most important emerging industrial firms.
- *Analysis of Preventive Behaviours of Rural Tourism Hosts in the Face of COVID-19 Pandemic: Application of the Health Belief Model* discusses preventive behaviors of rural tourism hosts and the COVID-19 utilizing the HBM model.
- *Investigating the Adoption of Precautionary Behaviours Among Young Rural Adults in South Iran During COVID-19* investigates the factors affecting youth intention and preventive behavior with respect to COVID-19, also using the HBM.

Besides of those the issue also discussed the following topics:

- Social, Environmental and Economic Impact Assessment of COVID-19 on Rural tourism.
- Dynamic conservation in risk society: A case study of COVID-19 pandemic risk in Kashan Qanat Irrigated Agriculture.
- Tourism development during the pandemic of coronavirus (COVID-19): Evidence from Iran.
- The impact of COVID-19 pandemic on food security, and food diversity of Iranian rural households.
- How do collective efficiency and norms influence the social resilience of Iranian villagers against COVID-19? The mediating role of Social leadership.
- Impacts of COVID-19 pandemic on micro and small enterprises (MSEs): Evidence from Rural Areas of Iran.
- Developing a paradigm model for resilience of rural entrepreneurial businesses in dealing with the COVID-19 crisis; application of grounded theory in western of Iran.

We hope that the papers presented in this special issue will be useful and stimulating for further understanding of risks, vulnerabilities, and counter mechanisms available to deal with a wide range of health and economic shocks faced by rural households and to properly design and develop social safety nets.

Due to the good response from the researchers to this Research Topic, the journal has decided to initiative, the Volume 2 with the same editorial team. Further information can be found at: https://www.frontiersin.org/research-topics/41035/addressing-the-effects-of-covid-19-on-rural-areas-in-low-and-middle-income-countries-volume-2.

## Author contributions

All authors listed have made a substantial, direct, and intellectual contribution to the work and approved it for publication.

## Conflict of interest

The authors declare that the research was conducted in the absence of any commercial or financial relationships that could be construed as a potential conflict of interest.

## Publisher's note

All claims expressed in this article are solely those of the authors and do not necessarily represent those of their affiliated organizations, or those of the publisher, the editors and the reviewers. Any product that may be evaluated in this article, or claim that may be made by its manufacturer, is not guaranteed or endorsed by the publisher.

